# COVID-19: Epidemiological Situation of Argentina and its Neighbor Countries after Three Months of Pandemic

**DOI:** 10.1017/dmp.2021.90

**Published:** 2021-03-25

**Authors:** María Laura Ramírez, Sofía Mickaela Martinez, Carolina del Valle Bessone, Daniel Alberto Allemandi, Daniela Alejandra Quinteros

**Affiliations:** 1 Unidad de Investigación y Desarrollo en Tecnología Farmacéutica (UNITEFA), CONICET, Cordoba, Argentina; 2 Departamento de Ciencias Farmacéuticas, Universidad Nacional de Córdoba, Córdoba, Argentina

**Keywords:** Argentina, COVID-19, epidemiological situation, pandemic, public health

## Abstract

**Objective:**

In this work, in order to establish a better comprehension of the association between Argentina and its neighbor countries’ capacity, and COVID-19 burden during the first 3 months, different indicators were evaluated.

**Method:**

We analyzed the association between GHSI, INFORM index and COVID-19 burden (number of confirmed cases and deaths), also the number of tests, lethality and the stringency of Governmental policies were evaluated.

**Results:**

Uruguay, Paraguay, and Bolivia started earlier different prevention measures. The number of tests differs, as Chile is the 1 that makes more. Uruguay and Paraguay register fewer positive cases and deaths from COVID-19. The GHS index is led by Brazil, followed by Argentina, and then Chile. However, the INFORM index is led by Uruguay followed by Argentina, while Chile and Paraguay are on par.

**Conclusion:**

The countries that took preventive measures earlier and carried out a more tests are the ones that are obtaining the best results against COVID-19.

## Introduction

On December 31, 2019, China reported the detection of a new coronavirus infection with possible origin of the outbreak in a seafood market in Wuhan City.^1^ The pathology was defined as Coronavirus Disease 2019 (COVID-19) and caused by the Severe Acute Respiratory Syndrome Coronavirus 2 (SARS-CoV-2), which spread from Asia to all the regions of the world. Latin America was an exception until February 2020, when Brazil reported the first case.^2^ Within weeks, the neighbor countries also confirmed their first cases. The outbreak in this region seems to be about 4 weeks behind Western Europe and 2 weeks behind the United States and Canada.^3^ On January 30, 2020 the World Health Organization (WHO) declared the epidemic by COVID-19 as a public health emergency of international concern. After 12 days, with more than 118000 cases in 114 countries and 4291 confirmed deaths among patients with COVID-19, WHO decreed the outbreak of the disease as a global pandemic due to the alarming and rapid spread.^4^ As of June 3, 2020, these numbers have risen to more than 6000000 confirmed cases and 379941 deaths.^5^

Pandemic preparedness is different throughout the world; however, what is clear is that the region where Argentina and its neighbor-countries (Uruguay, Brazil, Paraguay, Bolivia, and Chile) are, is particularly vulnerable to a destructive outbreak.^6,7^ These geopolitical regions are marked in many cases by high poverty, low water access and sanitation, and distrust in public governance.^3^ There are many factors that can influence the course of an infectious disease outbreak. In 2014, based on the experience acquired through the Ebola outbreak, public, and private organizations worldwide met with the intention of knowing the situation that different countries present to face a pandemic and created the Global Health Security (GHS) index.^8^ This index was developed with the aim of gauging countries’ capacity to deal with infectious disease outbreaks. The GHS index evaluates different categories related to prevention of the emergence or release of pathogens, early detection and reporting for epidemics potential international concern, response to the spread of an epidemic, and the robustness and sufficiency of health system to treat the sick and protect the health workers, among others. It is believed that the GHS index will spur measurable changes in national health security and improve international capability to address 1 of the world’s most omnipresent risks: infectious disease outbreaks that can lead to international epidemics and pandemics. The GHS index seeks to illuminate the gaps between disparities in capacity and inattention to biological threats in order to increase both political will and financing to fill them at the national and international levels.^8^ Unluckily, political will for accelerating health security is caught in a perpetual cycle of panic and neglect. The index ranges from 0 to 100, and assesses 6 main elements: prevention, detection and reporting, response, health system, compliance with norms and risk of infectious disease outbreaks. A higher GHS index indicates better preparedness.^9^

Likewise, the Joint Research Center (JRC) of the European commission has developed an index for risk management named “INFORM.”^10^ This index is a composite indicator that identifies countries at risk of humanitarian crisis and disaster that would overwhelm national response capacity. INFORM has been developed to improve the common evidence basis for risk analysis so all the entities involved can work together.^10^

In this work, the epidemiological situation of Argentina and its neighboring countries (Uruguay, Brazil, Paraguay, Bolivia, and Chile) from the first confirmed case (March 3, 2020), for up to 3 months is described, and discussed with the aim of contributing to the study of the policies and strategies carried out by each country to mitigate the consequences of the pandemic focused on preventive methods to prevent the pandemic from getting worse.^11^ In each country, in particular, the sanitary measures carried out, and the results obtained to date are evaluated. This data was associated with the effective preparedness by countries (defined by the HGS and INFORM indices) and with the right government responses for a better discussion of the results.

## Methodology

The source of information that has been used to carry out this work has been provided by the website of the Ministry of Health of Argentina,^12^ Brazil,^13^ Bolivia,^14^ Chile,^15^ Uruguay,^16^ and the Ministry of Public Health and Social Welfare of Paraguay.^17^ Country-level data on COVID-19 as at June 3, 2020 were also sourced from the “Worldometer.”^18^

Global data and official recommendations on COVID-19 were taken from the WHO official website.^19^ The GHSI and INFORM database were also consulted.^8,10^ Based on this official information, the epidemiological situation of Argentina and its neighbor counties in face of the COVID-19 pandemic is discussed.

## Results

### Current epidemiological situation in Argentina and neighbor countries

The emergence of a new infectious disease always implies a complex situation, especially if it occurs as an epidemic of significant extent or severity. The extent to which such a pandemic progresses, depends on many factors, some of which may be known, and some of which may not. Hence, the aim of the GHS index is to contribute to having safer and secure countries by giving access to information about their country’s existing capacities and plans to all the population for better preparation for a pandemic. With respect to the countries discussed in this work, Brazil tops the GHS index list closely followed by Argentina and Chile, respectively. Uruguay, Paraguay, and Bolivia follow in the order listed (Table 1). The average overall GHSI score totals 40.2, and in this sense Bolivia and Paraguay seems to be less prepared.^8^

**Table 1.** Summary of the epidemiological situation in Argentina compared to neighboring countries

Country	Date 1º confirmed case by COVID-19	Date 1º death by COVID-19	Date 1º preventive action (obligatory isolation, type of action)	Initial date of community transmission	% Lethality rate by COVID-19 (as of May-25)
***Argentina***	Mar-03	Mar-07	Mar-20 (strict and obligate in phases)	Mar-23	3.69 %
***Uruguay***	Mar-13	Mar-28	Mar-17 (not obligatory)	-	2.79 %
***Brazil***	Feb-26	Mar-17	-	Mar-20	6.26 %
***Bolivia***	Mar-10	Mar-28	Mar-12 (obligatory in phases)	Mar-14	3.92 %
***Chile***	Mar-03	Mar-20	Mar-18 (obligatory in phases)	Mar-16	1.03 %
***Paraguay***	Mar-07	Mar-20	Mar-20 (Total and obligatory)	Mar-20	1.27 %

INFORM index, as previously described, ranks countries based on their likelihood of requiring global assistance, evaluates the risk profile of each country, and enables trend analysis. The rank established for the countries evaluated in this work is: Uruguay > Argentina > Chile = Paraguay > Brazil > Bolivia (Table 1).^10^ The INFORM index can provide different types of information. One of them is the ranking of countries, where a qualitative evaluation of the current situation of each country is made. These results can be grouped into five classes: *very low*, *low*, *medium*, *high*, and *very high* risk of humanitarian crisis. Of the components, namely vulnerability and lack of coping capacity, are particularly important to the COVID-19 pandemic. Vulnerability dimensions the strength of the population to a crisis situation, and the lack of coping capacity considers factors of institutional strength like inadequacy of resources to alleviate the impact of a pandemic.^20^ Concerning vulnerability, Argentina, Uruguay, and Chile had *very low* scores but Brazil, Bolivia, and Paraguay record a *low* level. Regarding the lack of coping capacity, Uruguay tops the rank with a *very low* level while Argentina, Brazil, and Chile had *low* level, and Paraguay and Bolivia had *medium* levels.^10^

In addition to the previous indexes described, different indicators can also help to clarify the epidemiological situation of the countries. Some of them will be presented below and separated based on the different countries evaluated. An extended evaluation of Argentina´s situation is done in order to contribute to the state of the art.

#### Argentina

Founded on the information provided by WHO at the beginning of January about an infectious disease that has begun to spread in several eastern countries, in Argentina, on January 25, 2020, the Ministry of Health established an epidemiological vigilance protocol for hospitals and private clinics.^21^ The main objective of epidemiological vigilance under the COVID-19 framework is to quickly detect cases, provide adequate care to patients and implement research, prevention, and control measures in order to reduce the risk of propagation of the infection.^22^

The total number of people infected with COVID-19 is not known. All that is known is the infection status of those who have been tested. So far, Argentina has carried out 183862 COVID-19 tests, which represent 3.83 tests per 1000^18,23^ From these results, an average of 20 daily confirmed cases per 1000000 people are confirmed.^24^ The development of the *NEOKIT COVID-19*, by Argentine researchers and its approval by the National Administration of Medicines, Food and Medical Technology will allow an increase in the number of tests performed.^25^

**Figure 1.**
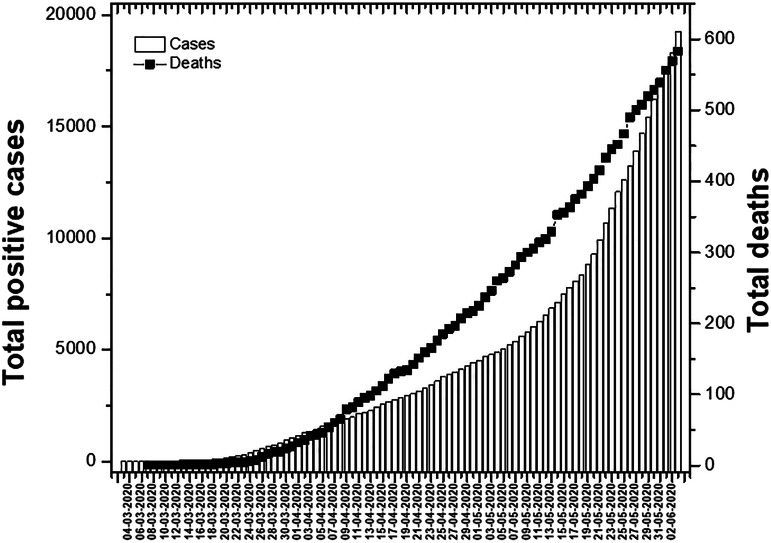
Current number of total cases confirmed by COVID-19 (bars) and total deaths (squares) from the beginning to the date in Argentina.

The first case of COVID-19 in Argentina was on March 3 by a patient that had returned from Italy, where a significant outbreak was ongoing.^26^ As at that date, the number of cases of infected people had been increasing, with a total number of confirmed cases reaching 18306 by June 3, 2020 (Figure 1). From this value, 50.56% corresponds to men, 49.09% to women, and 0.36% to others. Regarding the distribution of transmission in the country, positive cases have been registered in Autonomous City of Buenos Aires and 21 of the 23 Argentine provinces.^12^

Taking into account the fact that the cases initially reported were due to imported contagions, on March 14, the Government announced the restriction of entry of foreigners through land borders, and forbade for 30 days the entry of foreigners who have transited over risk countries in the last 14 days. Subsequently on March 20, it established Social, Preventive and Obligatory Isolation initially until March 31, but it was then extended 5 times, until June 7.^27,28^ Despite the preventive measures, on March 23, the first community contagion by COVID-19 was registered.^29^

The stages of the period of Social, Preventive, and Obligatory isolation established by the Argentine government are listed and detailed below:

- *Phase 1:* Strict isolation, from March 20 to April 13, 2020.
- *Phase 2:* Managed isolation, established from April 13 to 26, 2020.
- *Phase 3*: Isolation by geographic segmentation, from April 26 to May 10, 2020.
- *Phase 4*: Isolation with progressive reopening, from May 10 to June 7, 2020.
- *Phase 5:* Last phase search to re-establish new normality. It is expected to happen from June 7, 2020.

By June 3, 2020 the provinces that were in phase 5 were Corrientes and La Pampa; most of the provinces continued in phase 4 except for Buenos Aires metropolitan area and Autonomous City of Buenos Aires that were in phase 3.

Regarding mortality from the pandemic in Argentina, on March 7, the Argentinian Ministry of Health confirmed the first death in the country and in Latin America from COVID-19.^30^ Over the days, the number of deaths increased registering a total of 569 deaths as at June 3, 2020 (Figure 1).^31^

The lethality rate for COVID-19 in Argentina as at June 3 was 3.11% (Table 1). The lethality for this disease increases with age, reaching its maximum in people who are more than 80 years. However, the number of confirmed cases shows a Gaussian behavior, with a maximum in the persons aged between 30, and 39 years (Supplementary Figure 1).

Finally, it is interesting to note that the number of people who recovered by June 3, 2020, was 5980 which represents 29.63% of the total number of infected populations with COVID-19 in Argentina.^18^

#### Uruguay

Since the pandemic began, Uruguay has decided to take a different position than the rest of the Latin American countries in the fight against the coronavirus. The government opted for a careful strategy, focused on fine-tuning between health, and economy; thus avoiding mandatory quarantine and appealing to the responsibility of the population.^32^

On March 13, the Uruguayan government declared a health emergency due to Coronavirus, after the confirmation of the first 4 cases of COVID-19. The first measures included borders being partially closed, and a strict control of attendance at schools, among others. Continuing with the appearance of new cases of COVID-19, on March 17, the Uruguayan government appealed to a non-mandatory quarantine but urged the owners of large commercial shops to close them preventively and provisionally (this excludes food shops and pharmacies). On April 22, 973 rural schools resumed face-to-face classes with voluntary assistance, except those located in the department of Canelones. Classes were not resumed in schools located in urban centers. Currently, Uruguay has processed 45777 tests for coronavirus, which represents 13.02 tests per 1000 people.^18,23^ Of that total, 44762 were negative, 1015 positive, and 23 of them died.^16^ This represents a 2.78% case lethality rate by COVID-19 (Table 1).^33^ The departments with active confirmed cases are: Artigas, Canelones, Cerro Largo, Maldonado, Montevideo, Rivera, Salto, and San José.^16^

#### Brazil

Brazil is the nation with the highest number of confirmed cases in Latin America, and the second in America,^2,34^ only behind the United States. It is 1 of the countries with the least restrictive measures. At the government level, there are 2 contradictory measures due to the different political positions: the actual president demands a return to normality, however, some ministers and governors defend social distancing. The federal governments of Sao Paulo and Rio de Janeiro suspended classes and banned massive events, and the Brazilian Ministry of Health, recommends prevention measures such as telework, avoiding crowds and social contact. However, there are no clear measures that reach the entire population of Brazil.^35^ On February 3, the Brazilian government declared a National Public Health Emergency due to coronavirus and all appropriate preventive measures are taken. On February 26, Brazil reported the first positive case,^2^ and on March 17, the State of São Paulo registered the first death from COVID-19 in Brazil. Subsequently, on March 20, Brazil declared the state of community transmission by coronavirus (COVID-19) throughout the national territory. Currently, Brazil records 584016 confirmed cases,^24^ and 32548 deaths,^33^ with more than 100000 patients recovered. The number of tests that have been carried out only by public laboratories in the country as at May 29, 2020, the last date reported by the government, was 485000.^36^ This represents 2.28 tests per 1000 persons carried out.^23^ This country has a current case lethality rate of 5.61% for COVID-19 (Table 1), being the second country worldwide with more positive cases due to this pandemic.^18^

#### Bolivia

On February 2, 2020, the Bolivian government created the Emergency Operational Committee to detect possible cases of COVID-19, which included WHO officials and different ministries and entities specialized in health. The first 2 cases of coronavirus were reported on March 10.^37^ Subsequently, the government’s first measures were initiated. On March 12, 1 of the first actions carried on was the suspension of educational activities at all levels and the cancelation of European flights. On March 22, the government declared a state of health emergency by COVID-19, whose duration was scheduled up to April 30, 2020, but was extended until May 10 to subsequently apply the “dynamic quarantine.” On March 28, the first deaths were recorded. The lethality of COVID-19 in Bolivia as of June 3 is 3.42%, with 11638 confirmed cases^24^ and 400 deaths.^33^ Since the number of tests performed is not reported, we approximate the value as the sum of the number of confirmed and negative tests, which as at June 3 is around 34000 tests.^23^ The number of tests informed per 1000 people is 2.78.^18^

#### Chile

The first confirmed case of COVID-2019 in Chile was on March 3, 2020.^38^ Based on this first verified case, the outbreak spread throughout the country, reaching the 16 regions. As of June 3, 2020, 113628 confirmed cases^24^ and 1275 deaths^33^ had been recorded, with the case-fatality rate for this disease in the country at 1.09%, 1 of the lowest values in the region. On March 16, the Chilean health minister declared COVID-19 in phase 4, which implies that there is viral circulation and community spread of the disease. The president decreed a state of catastrophe for 90 days, 2 days later and decided to close the borders for the transit of foreign people. Currently, Chile is the country that performs the most PCR tests per 1000000 inhabitants in Latin America, with 628318 tests carried out as of June 3, 2020.^18^ Another important value to highlight is the number of tests actually done in Chile per 1000 people (32.87), which is the highest with respect to the evaluated countries.^23^ From March 22, the national curfew was decreed between 10:00 PM and 05:00 AM to reduce social contact and facilitate the inspection of people who must comply with mandatory quarantine. Currently, there is a total quarantine in several communes in the country which have a high infection index, while others have already completed the quarantine.^15^

#### Paraguay

The first confirmed case of COVID-19 was reported on March 7, 2020 in Asunción.^17^ On March 10, 3 days later, the second case was confirmed along with 3 more cases, all of which were infected by the second case. Hence, the National Government took measures in this regard: suspending classes and all activities involving the agglomeration of people. On March 15, with 8 confirmed cases, the Government ordered the partial closure of borders and established a night curfew, restricting free transit from 8:00 PM to 4:00 AM. The next day, the health emergency was declared when a new case of COVID-19 was confirmed.^17^ On March 20, 2020, the first death and the first case of community transmission in the country were confirmed. Because of this, the government decided to tighten sanitary measures, with a total quarantine that was extended successively until May 3, where an “intelligent” quarantine began with the opening of certain economic sectors under strict measures, divided into several phases. Currently, Paraguay registers 1070 confirmed cases^24^ and 11 deaths,^17,33^ with a lethality of 1.09% and 33081tests carried out.^23,39^ An average of 4.64 tests per 1000 people have been carried out.^18^

### Discussion of Argentine epidemiology and neighboring countries

**Figure 2.**
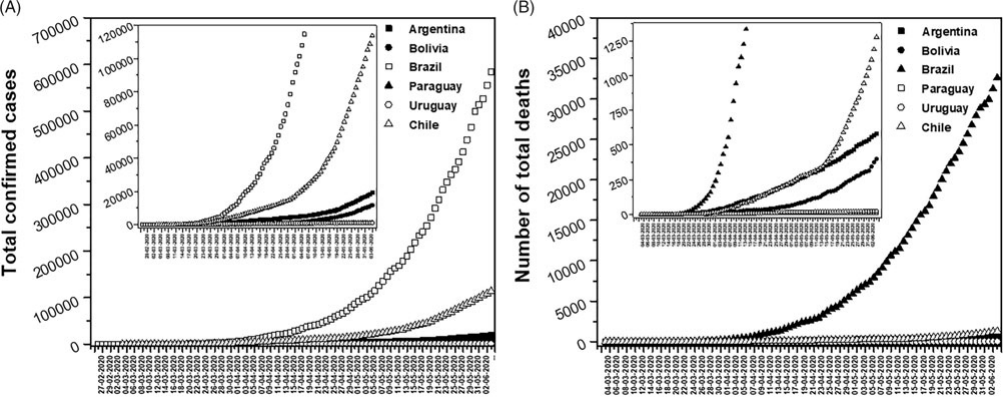
**A)** Evolution of the number of total confirmed cases of COVID-19 in Argentina and neighboring countries **(B)** Evolution of the number of total confirmed deaths from COVID-19 in Argentina and neighboring countries.

The first country that reported a confirmed COVID-19 case was Brazil on February 26, 2020 (Figure 2A). A few days later, neighboring countries such as Argentina, Chile and Paraguay began to report some cases until they spread throughout Latin America. The first death in the region was reported by Argentina on March 7, 2020, and by the end of the month all neighboring countries had also reported it. (Figure 2B).

The exponential increase in infections and deaths are the result of the so-called “community transmission,” a reality led by Bolivia on March 14 and promptly assumed in almost all of the aforementioned countries, with the exception of Uruguay (Table 1). Given the rapid advance of the pandemic and the different realities of the countries, the governments began to take different prevention and epidemiological surveillance measures. In this regard, Uruguay led the way in taking actions since it closed its borders on the same day the first case was confirmed. The next day, it established the closure of schools. Bolivia took a similar schedule and closed the schools and canceled European flights 2 days after the first positive test. In line with the above, Paraguay closed the education system 3 days after the first case was detected. However, Chile and Argentina took similar measures 12 and 17 days after, respectively. As of today, Brazil has not taken firm containment measures. Only on 27 March, the national government announced a temporary ban on foreign air travelers. Because of this, some regions have decided to impose local quarantines. Regarding isolation, Bolivia and Chile are the first governments to establish the mandatory quarantine by region. Subsequently, with the increase in positive cases and the registration of the first deaths from this virus, on March 20 Argentina and Paraguay decreed total, and mandatory isolation. This was not the case for Uruguay and Brazil, who have not established a mandatory isolation measure up to now (Table 1).

**Figure 3.**
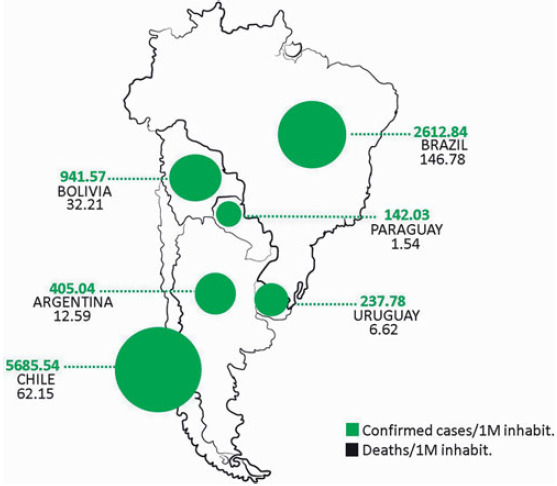
Summary map of Argentina and neighboring countries. The updated values of confirmed cases and deaths from COVID-19 per 1000000 (1M) inhabitants are detailed for each country.

It is important to highlight that the number of tests performed by each country is not the same. The confirmed cases are those who have a lab-confirmed infection, so the counts of confirmed cases depend on how much a country actually tests. Currently, Chile is the country that performed most PCR tests per millions of inhabitants in Latin America, Uruguay and Paraguay continue the ranking (Figure 3). The order of the other countries depends on the sources consulted, but in all the cases Argentina, Brazil and Bolivia are the countries that test less.

The Royal National Academy of Medicine defines lethality as the quotient between the number of deaths due to a certain disease in a period of time and the number of people affected by the same disease in the same period.^40^ This indicator can give an idea of the response capacity of the health system of the different countries of the region. Among the countries that best face this situation are Paraguay and Chile, with 1.09% lethality to COVID-19, they are followed by Uruguay (2.78%), Argentina (3.11%), Bolivia (3.42%), and finally Brazil with 5.61% (Table 1). Again, this indicator depends on the number of confirmed cases which also depends on the quantity of tests performed. Maybe it is important to evaluate as well the mortality that COVID-19 is causing. Mortality is defined as the number of deaths in a particular situation or period of time.^41^ The pandemic has left up to 379941 deaths all around the world.^18^ In the evaluated countries in this work, Brazil leads the list, followed by Chile and Bolivia; Argentina, Uruguay and Paraguay complete the list respectively (Table 1).

After this in-depth analysis, we can estimate the countries that seem to be doing better are Chile, Uruguay, and Paraguay. The last 2 nations are among those that first established the preventive arrangements, and this measure allows the contention of the evolution of COVID-19 pandemic. In addition, better preparation of the heath system was possible. Chile, however, began implementing containment measures some time later. These measures were accompanied from the beginning with a high test rate, which allowed them to control the situation. Uruguay and Paraguay also actively test their population. Mass population testing also makes it possible to detect asymptomatic patients. These actions allowed these 3 countries to better understand the pandemic and respond appropriately. Currently, Uruguay, and Paraguay are some of the Latin American countries that register fewer positive cases and deaths from COVID-19, maintaining greater control over the proliferation of the virus. It is also worth noting that these countries demographically and territorially, have the possibility of carrying out all these types of measures. They are geographically small territories with a low condensed population.

## Conclusion

The rapid advance of the COVID-19 pandemic in Argentina and in neighboring countries has mainly meant a health and economic crisis. The epidemiological behavior of each country was influenced by the public health policies, and the sanitary and preventive measures taken by each particular government. In Argentina, there is currently community transmission of the virus, which is why strict preventive sanitary measures are applied with the extension of preventive and compulsory isolation, sectored by region. This measure is estimated to have prevented the exponential growth of the pandemic in the country, making possible a better preparation of the health system for the future. Currently the number of infections is increasing, as well as the number of deaths. The need for an increase in the number of tests is widely discussed in order to effectively and quickly detect possible suspected cases, and take the necessary actions. We estimate that this is the reason why Argentina is not in the leading part of the list. In this regard, it is worth noting the recent and rapid progress in the development of new treatments, as well as test kits for COVID-19, by Argentine researchers. With respect to the neighboring countries, the epidemiological situation of some of them is better, such as Uruguay, Chile, and Paraguay, while in the case of Brazil it is more difficult. While awaiting effective treatments or vaccines, which are currently in the development stages, the only effective alternative is to adopt and implement all prevention and health containment procedures and appeal to social responsibility, to face this health problem worldwide. Finally, we remember that knowing the risks, however, is not enough. Political will is needed to protect people from the consequences of pandemics, to take action to save lives, and to build a safer and more secure world.

## Supplementary material

For supplementary material accompanying this paper visit http://dx.doi.org/10.1017/dmp.2021.90. click here to view supplementary material
