# Supplementary material for: COVID-19: Epidemiological Situation of Argentina and its Neighbor Countries after Three Months of Pandemic
Source: Disaster Med Public Health Prep. 2021 Mar 25:1–7. doi: 10.1017/dmp.2021.90 (PMC8193186; doi:10.1017/dmp.2021.90)
Supplement: Supplementary file 1 [file dmpsup.zip › S1935789321000902sup002.docx]

*Legends:*

***Figure 1.*** Currentnumber of totalcases confirmed by COVID-19(bars) and total deaths (squares) from the beginning to the date in Argentina.

***Figure 2. A)***Evolution of the number of total confirmed cases by COVID-19 in Argentina and neighboring countries.**B)**Evolution of the number of total confirmed deaths from COVID-19 in Argentina and neighboring countries.

***Figure 3.*** Summary map of Argentina and neighboring countries. The updated values for each country of confirmed cases and deaths from COVID-19 per one million (1M) inhabitants are detailed.

***Supplementary Figure 1.*** Summary of confirmed cases of COVID-19 and degree of lethality according to age groups in Argentina.
