# Supplementary figures and images for: COVID-19: Epidemiological Situation of Argentina and its Neighbor Countries after Three Months of Pandemic
Source: Disaster Med Public Health Prep. 2021 Mar 25:1–7. doi: 10.1017/dmp.2021.90 (PMC8193186; doi:10.1017/dmp.2021.90)

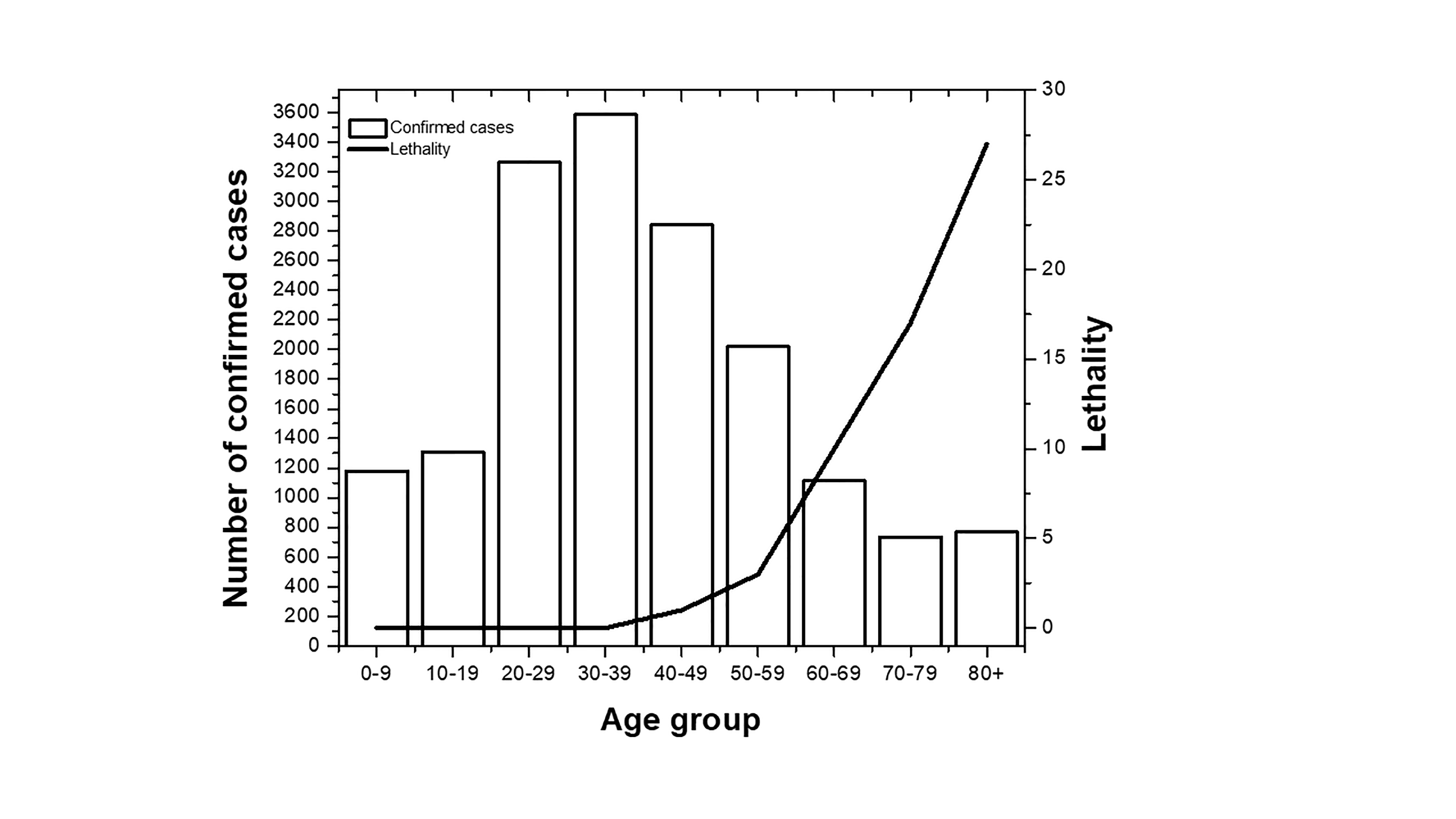

Supplement: Supplementary file 1 [file dmpsup.zip › S1935789321000902sup001.tif]
